# Is more psychotherapy a dead horse? An essay on the (in)effectiveness of individual treatment for mental suffering

**DOI:** 10.1371/journal.pmen.0000194

**Published:** 2024-12-05

**Authors:** Laura Batstra, Sami Timimi

**Affiliations:** 1 Department of Child and Family Welfare, University of Groningen, Groningen, Netherlands; 2 Child and Adolescent Mental Health Service, Lincolnshire Partnership NHS Foundation Trust, Lincoln, United Kingdom; PLOS: Public Library of Science, UNITED KINGDOM OF GREAT BRITAIN AND NORTHERN IRELAND

## Abstract

In the past decades, psychological and pharmacological treatment access has improved, but the prevalence of mental health conditions has nevertheless increased across all age groups, and particularly in young people. Recent reviews, taking biases and quality of included studies into account, confirm the relative ineffectiveness of individual psychotherapies for alleviating mental suffering. Many new forms of individual therapy have been developed since the 1970s, without resulting in improved rates of recovery from treatment. Various stakeholders keep advocating for more psychotherapy, but instead, we propose that more primary prevention strategies may be our best hope for reducing the burden of mental disorders.

## Introduction

The tribal wisdom of the Dakota Indians, passed on from one generation to the next, says that when you discover that you are riding a dead horse, the best strategy is to dismount. However, in modern business, because heavy investment factors are taken into consideration, other strategies are often tried with ‘dead horses’, including reclassifying the dead horse as "living-impaired" and providing additional funding to increase the dead horse’s performance [1].

At present, an estimated 1 in 8 adults [2] and more than 1 in 10 youths [3] around the world meet criteria for a mental disorder (as defined by classification manuals such as the Diagnostic and Statistical Manual of Mental Disorders (DSM)). According to a cross-national analysis of population surveys from 29 countries published in 2023, by the age of 75 years, approximately half the population will have endured one or more mental health conditions, which typically first emerged in childhood, adolescence, or young adulthood [4]. In these surveys, the Composite International Diagnostic Interview of the World Health Organisation (WHO), a fully structured psychiatric diagnostic interview, was used to assess age of onset and lifetime prevalence of mental health conditions. Studies using self-report questionnaires suggest even higher prevalence numbers. For instance, a survey in 2019 in the UK of one thousand young people found that 68% thought they have had or are currently experiencing a mental health problem. In addition, it revealed that there had been a 45% increase in mental health referrals of under-18s in the previous two years [5]. Another 2019 paper using a child self-report questionnaire methodology, came up with a prevalence figure for mental health problems in 11- to 15-year-olds of 42% [6]. A 2020 study from New Zealand reported that 86% of people will be eligible for a psychiatric diagnosis by the time they’re 45 years old, with about half of the population having met the criteria for a ‘disorder’ by the age of 18 [7].

These findings are consistent with widely reported declines in mental health in the last few decades. International studies show this decline in mental health across all age and gender groups, with English-speaking countries having the lowest levels of mental well-being, and the 18–24 age group having the worst mental health of all age groups [8].

In response to these alarming figures, many scholars call for better detection and more (early) interventions [3, 4], with psychotherapies and pharmacotherapies often recommended as first line treatments [9]. We acknowledge that these interventions benefit some individuals. In this essay however, we challenge the call for more interventions aimed at individuals and make a plea for a radical paradigm shift in mental health practice, research, and policy.

### The treatment prevalence paradox

In the past decades, the global mental health market size rapidly increased to US$ 435.2 billion in 2023 and is expected to grow further [10]. While more is spent on treatment of individuals, the general population prevalence of conditions is still on the rise [11] and, if current practice continues, will likely keep growing [12]. Hence, on the one hand treatment access has improved and become more available since the 1980s, but on the other hand the omnipresence of mental health conditions has not decreased, but instead has increased across all age groups, and particularly in young people. Ormel and colleagues [13] evaluated several explanations for this so-called Treatment Prevention Paradox (TPP). They found little evidence for the hypothesis that more awareness and greater willingness to report mental problems increased the detection of epidemiological prevalence. Instead, next to possible iatrogenic effects of current treatments, substantial gaps in the quality of implementation of treatment protocols in real-world settings, and biases and overestimations of treatment efficacy in the published literature, likely account for most of the TPP.

In line with the latter explanation, while in 2014 [14] a comprehensive review of meta-analyses of both psychotherapies and pharmacotherapies in mental health conditions reported a modest effect size of 0.50, an umbrella review by Leichsenring and colleagues in 2022 [9] using placebo or treatment as usual (TAU) as comparison groups (instead of less optimal waiting lists) and including a formal assessment of risk of bias and quality of studies, yielded much lower effect sizes of 0.34 for psychotherapies and 0.36 for pharmacotherapies. The authors conclude that “After more than half a century of research, thousands of RCTs and millions of invested funds, the effect sizes of psychotherapies and pharmacotherapies for mental health conditions are limited, suggesting a ceiling effect for treatment research as presently conducted.”.

Compared to effect sizes based on continuous measures, binary outcome measures are easier to interpret and allow for a comparable outcome metric across different disorders, providing an indication of their relative ‘treatability’. Binary measures however, can over-estimate efficacy depending for example on how you define the cut-off between ‘recovered’ and not. In a meta-analytic study published in 2024 Cuijpers and colleagues defined absolute rate of response as ‘at least 50% symptom reduction between baseline and post-test in the treatment and control conditions’ and they examined these binary outcomes of psychotherapies for eight major mental disorders [15]. Analyses revealed that for seven out of eight disorders, approximately five persons have to receive psychotherapy to reach one beneficial outcome. The numbers needed to treat (NNT) when controlling for publication bias were even higher of between 5.3 and 17.6. Obsessive compulsive disorder (OCD) was an exception, with between 2.4- and 3.4-persons needing treatment for one person to benefit. This means that in at least 80% of psychotherapy interventions (with the possible exception of OCD) there does not appear to be any substantial problem reduction.

An NNT of 5 is usually interpreted as being on the borderline of what is still acceptable for the effectiveness of treatments [16]. Cuijpers and colleagues conclude their article with “More effective interventions, as well as therapies for those not responding to a first-line treatment, are clearly needed”. However, to what extent can we expect a development of more effective interventions for successfully treating mental suffering?

### No evidence for improved outcomes

There are over 500 different forms of therapy documented and every year new ones come on stream. Not only has this proliferation of models not resulted in improved outcomes, but studies show that psychotherapy is less effective for those who have a lower income, have minority status, or are on antidepressants [17–19]. Controlled trials that test efficacy of therapies have been conducted since the 1970s, but have not shown improved rates of recovery from treatment. Some comparisons even suggest outcomes from therapy in controlled trials have got slightly worse over time [20–24].

This means that technically speaking there is no evidence that the proliferation of psychological techniques to treat mental health conditions has resulted in improved outcomes for those who seek help. In most healthcare fields it’s possible to see gradual, and sometimes sudden, improvement in outcomes. Survival rates after heart attacks have been increasing, thanks to increased understanding of the physiology, leading to better treatments. Average cancer survival years have improved for most cancers, and vaccination programmes have reduced the prevalence and lethality of many diseases. That’s what happens when the more objective metrics of care are central to outcomes, something that, according to the above findings, cannot be assumed to be the case in mental health treatments.

If at most 20% of treated persons improve–often only temporarily [25]–and the development of more effective treatments is not to be expected, investing in individual treatments may not be the most effective use of a health budget.

### Intervention as prevention

Whilst individual treatments later in life are not as effective as we hoped, perhaps early detection and early intervention offer opportunities to find that illusive key to improving outcomes. As many conditions are thought to first emerge in childhood and adolescence [4, 7], the idea that serious psychological problems can be avoided by early identification and treatment of disorders is widespread [e.g., 26].

This ‘intervention as prevention’ hypothesis rests in a (bio)medical framing that locates problems *in* an individual. As discussed earlier, this model works well for many physical diseases with an identifiable pathogenesis. A serious bacterial infection is best identified as early as possible, so that a course of antibiotics can be given. When applied to mental health problems, this type of framework attributes mental distress and behavioural disturbances to some sort of dysfunction in the structure or functioning of the brain/mind. This is the paradigm that has dominated research and practice in health care in recent decades [27], even though many billions spent on brain research have not led to the identification of biomarkers for any mental health condition [28]. The chance that this will ever happen seems unlikely as human brains are too variable and multifaceted. The brain is the most complex known structure in the universe containing around 86 billion neurons, 85 billion other cells, and over 100 trillion connections [29]. Psychiatric classifications rely on vague definitions, which means that the presenting problems of people with the same diagnostic label may be very different from each other [30]. The assumption that research will one day reveal a similar pathophysiology within all the different psychiatric diagnostic categories, is as illogical as it is persistent. The temptation to turn something that defies certainty into a concrete entity is as culturally attractive as it is lucrative.

Human emotions and behaviours, especially those of children and adolescents, are far too complex, socially situated, and changeable to ‘catch’ with diagnostic categories and treat with corresponding pharmacological or psychological algorithmic interventions. It should therefore come as no surprise that there is little evidence to support the ‘intervention as prevention’ hypothesis. In a recent overview of systematic reviews Roest and colleagues concluded that long‐term benefits of psychosocial and pharmacological childhood interventions appear to be small at best, while harms of pharmacological and psychosocial treatments cannot be ruled out [31]. A study by Copeland and colleagues involving 1420 children followed long-term found that psychiatric treatment did not reduce the risk for adult emotional or substance use disorder, but in fact resulted in increasing the risk of substance abuse among participants who received treatment [32].

Like early treatment, early diagnosis of a DSM-disorder may also do more harm than good. Evidence for this worrying hypothesis comes from two studies on the effects of early DSM-diagnosis, in this case of ADHD. A publication in 2020 as part of the National longitudinal study of Irish children, examined the sociodemographic, clinical, and psychological variables that differentiate children with high hyperactivity/inattention behaviours, who had and had not received a diagnosis of ADHD. By age 13, those who had held an ADHD diagnosis at 9 years showed more emotional and peer relationship problems, worse prosocial behaviour, and poorer self-concept, than those who did not receive a diagnosis despite similar levels of ‘symptoms’ at 9 [33].

A similar study, this time as part of the Longitudinal Study of Australian Children, included a comparison of nearly 400 children diagnosed with ADHD, matched to a group who had similar levels of hyperactivity/inattention but did not get an ADHD diagnosis. By 15 years old (an average follow-up of 7 years) those with the diagnosis were doing worse on a number of variables including self-harm, feeling that they could succeed academically, sense of self-efficacy, and demonstrating negative social behaviours [34]. Both of these studies suggest that in the long term a child may be better off not getting a diagnosis of ADHD even if it shows all the behaviours that may lead to a diagnosis. It seems preferable to speak in terms of variation and difference than in terms of deviation and disorder.

Findings of this sort are seriously disturbing in terms of ethics of child welfare and child rights. Research on the usefulness of individual diagnoses and treatments should have been conducted well before millions of children worldwide were subjected to it. How did it get to this point?

### I’m not ill, I’m hurt

In the context of the increasing popularity of the biomedical model and pharmacological treatments in the 1970s and 1980s, the randomised controlled trial (RCT) became the standard method of evaluating drug treatments as well as psychotherapy. In order to be eligible for research funding, psychological interventions need to be standardized (i.e., manualized) and aimed at reducing the ‘symptoms’ of a specific DSM-defined psychiatric diagnosis. While RCT’s maximize internal validity, ecological validity is seriously compromised because the delivery of a fixed number of therapy sessions to a carefully selected relatively homogeneous group without comorbidities in close adherence with a step-by-step manual, bears little resemblance to routine clinical practice. In addition, sexual, gender, and racial/ethnic minorities are often under-represented in these studies, creating an absence of data about mental health treatment for many minority groups.

Next to ecological validity issues, the failure to advance clinical care may also in part rest on a lack of attention to socioeconomic determinants of mental suffering and the inability to move beyond the medical model of diagnosis and treatment [35]. Research into social determinants requires a different design than the RCTs popular with funding agencies. More than 10 years ago, counselling psychologist Paul Moloney published his book *The Therapy Industry*: *The Irresistible Rise of the Talking Cure*, *and Why It Doesn’t Work* [36]. “I’m not ill, I’m hurt”, words from Scottish mental health service user David Adam, is the title of a chapter about the hidden injuries of social inequality. There is compelling evidence that the risk of developing mental suffering is linked to life circumstances such as living in poverty, loneliness, racial discrimination, housing disadvantage and other markers of social disadvantage [37]. How is talking to a professional who is (sometimes well) paid to listen attentively going to solve any of these problems? According to Moloney therapy locates the problem within the individual, allowing society to look the other way and present mental disorder as an individual weakness or vulnerability, rather than a consequence of living a marginalised existence in an unequal society.

In recent decades, we have increasingly passed the buck of societal problems to children, who are the ones most affected by unfortunate circumstances and lack the power to say no to stigmatizing psychiatric labels and treatments. For example, children growing up in poverty [38] and/or facing racism [39] are much more likely to have a mental disorder diagnosis than their contemporaries. What do these children and adults with mental suffering really need? Probably not more therapy.

### Paradigm shift

In a PLOS blog entitled ’Less research is needed’, Trisha Greenhalgh [40] described how scientists sometimes fail to let go of their once promising but proven hopeless research ideas. They keep saying that more funding is needed because “However large the trial, the answer always seems to lie just around the next empirical corner”. Indeed, Cuijpers and colleagues found in their systematic review and meta-analysis that psychotherapy does not lead to significant improvement for at least 80% of people receiving it, and subsequently conclude their article with “More effective interventions are clearly needed” [15]. Roest and colleagues in their umbrella review revealed the absence of scientific support for the long-term effectiveness of childhood treatments. A group of researchers and developers of such therapies wrote a response to this conclusion and argued, perhaps not surprisingly, that there *is* evidence for beneficial longer term treatment effects and that evidence-based treatment should continue to be offered to children with mental health conditions [41], without providing much empirical support for these opinions [42].

Calling for more diagnoses and treatments is like riding a dead horse. Letting go of the horse, that is letting go of the idea that therapy is *typically* the best answer to mental suffering, will be a difficult challenge because of the many intellectual and financial vested interests of, for example, the therapy industry, pharmaceutical manufacturers, policy makers, academics, training developers and patient interest groups. However, less focus on individual therapy may make room for primary prevention addressing the long neglected social determinants of mental ill health.

### Recommendations

We conclude with three recommendations that may boost the much-needed paradigm shift away from more psychotherapy:

1. Invest in primary prevention
  In the past decades, approaches to prevention have been inequitably prioritized, with the majority of available resources devoted to (research into) secondary and tertiary prevention strategies: retrospectively trying to alleviate the damage of adverse living conditions and traumatic experiences with individualised interventions [37]. Despite all efforts, the prevalence of mental health conditions worldwide has increased, and is expected to keep growing. Primary prevention strategies may be our best hope for reducing the burden of mental distress that afflict so many individuals and societies at large. With more political focus on equality, social justice, inclusion, living conditions and so on, fewer people will need to rely on treatments of dubious value in such unequal societies. The Treatment Prevalence Paradox shows us that mental health services are mopping up with the tap open.
2. Save individual treatments for the most severe problems
  We are not calling for individual treatments to be abolished. However, it seems important to allocate psychotherapy resources to those who need it the most. Simply not treating any mild problems would be suboptimal since some will eventually progress into more severe suffering. Stepped approaches [43] aim at reducing the provision of unnecessary and possibly harmful interventions without risking neglecting or minimising more severe mental health presentations. They are based on the view that mental struggles are a part of human existence and sometimes provide an important signal to, for example, slow down or organize life differently. Stepped treatment starts with first-line health care efforts that are the least intensive of those available but still likely to provide a significant health gain. Continuous assessments of the helpseekers’ condition are crucial–with timely stepping up if a lesser intervention is not sufficiently improving mental health. By carefully following steps like normalising, de-medicalising, psycho-education, watchful waiting, supportive conversations and autonomy-enhancing minimal interventions, specialized treatment may be postponed or no longer necessary. Specialist care is then reserved for the more serious and/or persistent problems. Even here though a paradigm shift away from imagining there are technical fixes to be delivered, allows for more context rich and relationship centred models of treatment to be used.
3. Acknowledge the value of qualitative research
  Investigating the possibilities of stepped care models does not fit the popular RCT design. RCT’s can only test already existing hypotheses, whilst qualitative designs make it possible to explore new ways of thinking and acting. This is vital for developing new paradigms of care. In addition, qualitative research can do more justice to the pluralization of our life worlds and the intersectionality of minority groups [44], which may not be represented in RCT’s with standardized treatment protocols [e.g.^45^]. Hence, if we want less (unnecessary) *and* better psychotherapy treatments, we need more qualitative research.

By working towards practices that safely deliver on the above recommendations, we are ensuring that we help to proactively combat mental suffering as opposed to leaning too heavily on psychotherapy in a reactive manner, when, at the end of the day, psychotherapy is not therapeutic for all.
